# Delayed Onset of Pleural Effusion After Thoracic Radiation Therapy for Hodgkin Lymphoma: A Case Report With Over 30-Year Follow-Up

**DOI:** 10.7759/cureus.27138

**Published:** 2022-07-22

**Authors:** Atsuto Katano, Masanari Minamitani, Hideomi Yamashita, Keiichi Nakagawa

**Affiliations:** 1 Radiology, The University of Tokyo Hospital, Tokyo, JPN; 2 Department of Comprehensive Radiation Oncology, The University of Tokyo, Graduate School of Medicine, Tokyo, JPN

**Keywords:** late complications, lung-injury, pleural fluid (pf), radiation-induced lesion, adult hodgkin lymphoma survivor

## Abstract

Pleural effusion after thoracic radiation therapy is an important adverse event affecting the patient's quality of life. A 58-year-old woman was referred to our hospital with the complaint of exertional dyspnea for several months. Chest radiography revealed left pleural effusion, most likely related to radiotherapy to mediastinal and left cervical lymph nodes in the treatment of localized Hodgkin lymphoma 25 years ago. She was followed for the next eight years and experienced a slow exacerbation of pleural effusion. Here, we report a case of extremely late-onset pleural effusion after thoracic radiotherapy.

## Introduction

Hodgkin lymphoma is a malignant B-cell lymphoma that is histopathologically characterized by the presence of Reed-Sternberg cells. Localized Hodgkin lymphoma is classified into favorable and unfavorable prognostic groups based on several risk factors [1]. Since a favorable prognosis of early-stage Hodgkin lymphoma implies a long-term survival rate, there has been interest in the clinical management of late events that occur after curative treatment [2]. For example, mantle irradiation alone has been utilized for early-stage Hodgkin lymphoma and has an excellent clinical outcome, with a 10-year overall survival rate of 98.2% [3]. However, normal tissue damage caused by radiation therapy results in delayed toxicity, such as in the case of cardiovascular diseases and secondary cancers [4].

Pleural effusion after chest radiation therapy is also an important side effect affecting a patient's quality of life, causing chest pain, nonproductive cough, orthopnea, and dyspnea [5]. In this report, we describe a case of pleural effusion that occurred 25 years after thoracic radiotherapy.

## Case presentation

**Figure 1:**
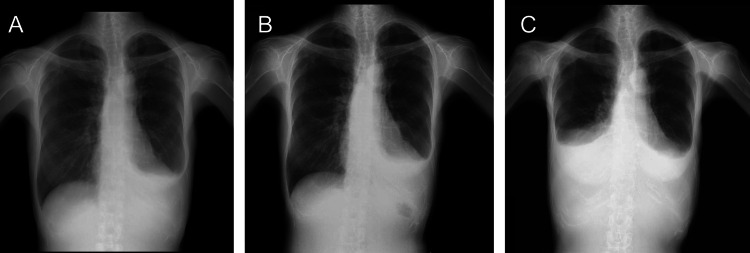
Chest radiography images Chest radiography images taken (A) 25 years, (B) 27 years, and (C) 33 years after thoracic radiation therapy for Hodgkin lymphoma.

A 58-year-old woman was referred to a pulmonologist at our institution with complaints of exertional dyspnea. She had a medical history of radiotherapy to the mediastinal and cervical lymph nodes, called mantle field irradiation, for the treatment of stage IIA Hodgkin lymphoma 25 years prior to the time of reference. Her medical history also included hypertension, hypothyroidism, hepatitis C following interferon treatment, and bronchial asthma. Chest radiography revealed left pleural effusion (Figure 1A).

**Figure 2:**
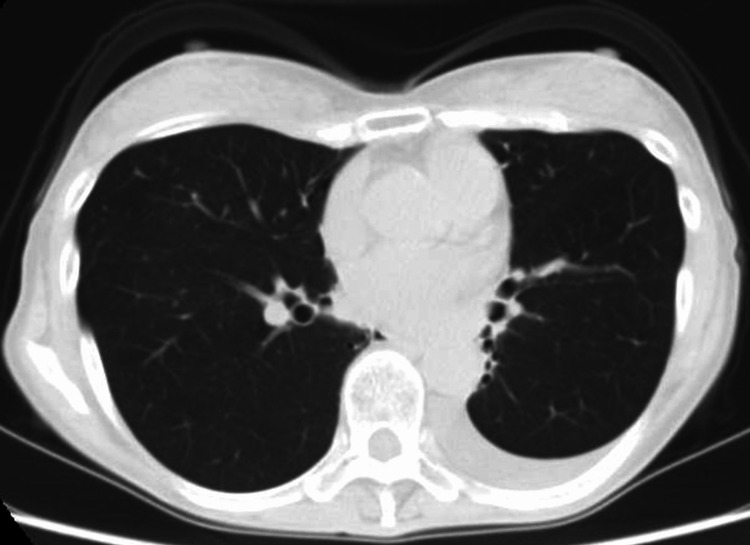
A computed tomography scan of the bronchi A computed tomography scan demonstrating that the bronchi in the left lung are tractioned toward the mediastinum, thus indicating decreased lung volume.

Computed tomography showed that the bronchi were tractioned toward the mediastinum, suggesting decreased lung volume caused by radiation-related fibrotic changes (Figure 2).

Thoracentesis was performed for diagnostic purposes and symptom palliation. Pleural fluid analysis revealed a total protein level of 4.5 g/dL, a glucose level of 112 mg/dL, lactate dehydrogenase level of 108 U/L, albumin level of 3.0 g/dL, and carcinoembryonic antigen of 1.4 ng/ml, whereas blood serum analysis revealed a total protein level of 7.2 g/dL and lactate dehydrogenase level of 223 U/L. According to Light's criteria for pleural fluid [6], the patient's pleural fluid was classified as exudate because the pleural fluid/serum protein level ratio was >0.5. Both pleural fluid smear and culture for acid-fast bacilli results were negative. No significant increase in adenosine deaminase or hyaluronic acid levels was observed. The pleural fluid cytology result was class 1 with no malignancy. She showed no liver dysfunction (aspartate aminotransferase of 22 U/L, alanine aminotransferase of 10 U/L, total bilirubin of 0.6 mg/dL, prothrombin time of 89%, albumin of 4.1 g/dL) and her hypothyroidism was well controlled (thyroid-stimulating hormone of 4.88 microIU/ml, free triiodothyronine of 1.7 pg/mL, and free thyroxine of 0.98 ng/mL). Echocardiography showed no obvious sign of heart failure with a good left ventricular ejection fraction (EF of 65%) and right ventricular function (right ventricular systolic pressure of 38 mmHg, right atrial pressure of 3 mmHg).

After two years, the pleural effusion on the left side gradually increased (Figure 1B), and she underwent thoracentesis once again for cytological examination, which resulted in no malignancy. After more than six years, the pleural effusion had exacerbated, presenting in the right chest (Figure 1C). Thoracentesis for cytological examination of the right chest revealed no malignancy (Table 1).

**Table 1:** Pleural fluid and serum analysis of the patient During the follow-up period, three thoracenteses were performed. No significant changes in their quantitative characteristics were observed. RT - radiotherapy

Characteristics	1st thoracentesis (25 years after RT)	2nd thoracentesis (27 years after RT)	3rd thoracentesis (33 years after RT)
Side of thoracentesis	Left	Left	Right
Pleural fluid			
Total protein (g/dL)	4.5	4.3	4.4
Albumin (g/dL)	3	2.9	2.9
Albumin/globulin ratio	2	2.07	1.93
Lactate dehydrogenase (U/L)	108	101	104
Glucose (mg/dL)	112	102	98
Carcinoembryonic antigen (ng/mL)	1.3	1	1.8
Serum			
Total Protein (g/dL)	7.2	7.3	7.8
Lactate dehydrogenase (U/L)	223	244	233
Characterization of pleural effusion assessed by Light's criteria	Exudates	Exudates	Exudates
Cytopathology	No malignancy	No malignancy	No malignancy

Treatment included thoracentesis to relieve respiratory distress, followed by the oral administration of tolvaptan, a competitive vasopressin receptor antagonist that functions as a diuretic, at 7.5 mg/day.

## Discussion

Here, we report a case of late-onset pleural effusion after thoracic radiotherapy. The production of pleural effusions after radiotherapy is caused by chronic pleuritis and lymphatic obstruction due to mediastinal fibrosis [7]. Aqeel et al. conducted a retrospective analysis of 96 cancer patients receiving thoracic irradiation, and more than half of the patients reported developing new pleural effusions with a median time to development of six months [8]. They also reported that 67% of the patients developed pleural effusion ipsilateral to the irradiated site. Zhao et al. reported that the incidence rate of radiation-inducing pleural effusion in lung cancer patients was 24.9%, and the median onset time from the end of thoracic radiotherapy was 3.7 months [9]. Compared to these values, the timing of the onset of pleural effusions we reported is characteristic. Shen et al. also reported a late onset of pleural effusion that occurred 25 years after radiotherapy for cervical lymphoma [10].

A consensus on the management of radiation-induced pleural effusion is not yet well-established. Its treatment is usually similar to that of general pleural effusion and includes diuretic medications, thoracentesis, an indwelling pleural catheter, and pleural sclerosis [11]. Kumagai et al. succeeded in the oral administration of 30 mg of prednisolone corticosteroid for pleural effusion due to radiation-induced pleuritis after chemoradiotherapy in a patient with esophageal cancer [12]. 

Currently, the standard treatment for early-stage Hodgkin lymphoma appears to be a combination of systemic chemotherapy and radiation therapy, with a trend toward smaller doses and more localized irradiation fields called involved-field irradiation that can avoid severe late adverse events [13]. Recently, Borchman and colleagues reported excellent clinical outcomes of radiotherapy-omitted treatment strategies using positron emission tomography assessment after receiving two cycles of dose-escalated bleomycin, etoposide, doxorubicin, cyclophosphamide, vincristine, procarbazine, and prednisone (BEACOPP) plus two cycles of doxorubicin, bleomycin, vinblastine, and dacarbazine (ABVD) [14].

## Conclusions

Our report describes an extremely late-onset case of pleural effusion that occurred 25 years after thoracic radiotherapy. Although the role of radiotherapy in early-stage Hodgkin lymphoma has been decreasing in recent decades, we have presented the possibility of late side effects in an era where large-field irradiation is more commonly used. Moreover, there is a lack of knowledge regarding the mechanisms by which radiotherapy affects the development of pleural effusions. Integrated studies involving clinical research, radiobiology, and physiology are important to reduce the risk of post-radiotherapy pleural effusions.
